# High intensity human activity limits ultra-high altitude soil microbial community dispersal and keystone taxa distribution in southeastern Tibetan Plateau

**DOI:** 10.3389/fmicb.2025.1666493

**Published:** 2025-10-03

**Authors:** Wenzu Liu, Hongfang Ma, Mengyao Dong, Yaning Song, Ruihong Wang, Zhuonan Hou, Daqing Luo, Heping Ma, Yuquan Wei

**Affiliations:** ^1^Institute of Tibet Plateau Ecology, Xizang Agricultural and Animal Husbandry University, Nyingchi, China; ^2^Beijing Key Laboratory of Biodiversity and Organic Farming, College of Resources and Environmental Science, China Agricultural University, Beijing, China; ^3^College of Grassland Science and Technology, China Agricultural University, Beijing, China; ^4^State Key Laboratory for Soil Erosion and Dryland Farming on the Loes Plateau, Institute of Soil and Water Conservation, Chinese Academy of Science and Ministry of Water Resources, Yangling, Shaanxi, China; ^5^University of Chinese Academy of Sciences, Beijing, China

**Keywords:** human footprint index, community assembly, environmental heterogeneity, microbial network, alpine regions

## Abstract

Increasing human activities have caused ecological damage to the environment, especially in the ecologically sensitive alpine regions such as the southeastern Tibetan Plateau. However, how different intensities of human activities in the alpine regions affect the community assembly process of soil microorganisms and the distribution of keystone taxa remains unclear. This study examines the relationship between human activity intensity and soil microbial dynamics in three different Human Footprint Index (HFI) regions. The microbial community structure and assembly processes were investigated within over 200 km from Gongbo’gyamda County to Bayi District in the southeastern section of Tibet. The results show that human activities changed the content of soil nitrogen (*r* = 0.50) and phosphorus (*r* = −0.46), which affected bacterial diversity (phosphorus for Sobs, *r* = −0.41, for Shannon index, *r* = −0.37; nitrogen for Shannon index, *r* = 0.37). Human activities increase the complexity of microbial networks but decrease the stability of soil micro-ecosystems. As elevtation increases, the dispersal limitation of microbial communities decreases (total effect size: bacterial = −0.705, fungal = −0.745). However, human activities directly or indirectly exacerbate this diffusion limitation (total effect size: bacterial = 0.488, fungal = 0.252). Keystone taxa are closely related to the assembly processes, which could significantly restrain the dispersal limiation of microbial community, especially fungal (*r* = −0.458). These insights help to understand the ecological impact of human disturbances on microbial networks and provide a basis for future conservation strategies aimed at mitigating biodiversity loss in fragile ecosystems like southeastern Tibet.

## 1 Introduction

The Qinghai-Tibet Plateau, recognized as a global biodiversity hotspot with extreme climatic conditions and ecological sensitivity, which becomes a critical natural laboratory for studying ecosystem responses to disturbances (Wu et al., 2022; Shen et al., 2018). In southeastern Tibet, intensified human activities (e.g., grazing, tourism, and infrastructure development) have led to habitat fragmentation, soil degradation, and biodiversity loss over recent decades, despite policy interventions aimed at mitigation (Hua et al., 2022; Li et al., 2021). Soil microorganisms play crucial roles in nutrient cycling, environmental remediation, and maintaining ecosystem stability (Bardgett and van der Putten, 2014; Falkowski et al., 2008; Hartmann and Six, 2023). However, the effects of varying human activity intensities on soil microbial communities in ecologically vulnerable regions at ultra-high altitudes in the Qinghai-Tibet Plateau were still underexplored, which limit our ability to assess ecosystem vulnerability and develop targeted conservation strategies.

The composition, diversity, and assembly of microbial communities undergo dynamic changes in space and time, and the assembly process has become a focus of microbial ecology research (Zhou and Ning, 2017; Jiao et al., 2020; Stanić et al., 2025). According to the niche theory, species characteristics, interspecific interactions and environmental factors determine the community structure, which reflects deterministic processes. In contrast, the neutral theory holds that all species have equal ecological functions, and the community structure is mainly driven by stochastic processes (He et al., 2025). Human activities significantly influence these assembly mechanisms by altering soil physicochemical properties, nutrient availability, and habitat heterogeneity (Kong et al., 2022; Hu et al., 2023). In agricultural intensifications, elevated soil pH and phosphorus levels enhance deterministic selection (Weigel et al., 2023), while low-disturbance areas favor stochastic dominance (Hu et al., 2023). Studies in some plateau regions have revealed that elevational gradients and land-use changes drive shifts in assembly processes, often through nutrient heterogeneity and environmental filtering (Fu B. et al., 2023; Fu F. et al., 2023; Yang et al., 2014; Chen et al., 2025). Another study on the Inner Mongolia grassland indicates that grazing significantly increases microbial beta diversity and enhances the proportion of deterministic processes (Xun et al., 2018). Mechanistically, activities like trampling and overgrazing cause mechanical compaction of soil, and livestock hoof prints will reduce soil porosity and increase bulk density (Byrnes et al., 2018), limiting microbial dispersal via habitat fragmentation, particularly for less mobile taxa (Hanson et al., 2012; Doherty et al., 2021). Despite these insights, the specific impacts of human activity gradients on microbial assembly in ultra-high altitude fragile ecosystems have not been reported.

Building on assembly processes, keystone taxa, highly connected taxonomic groups within microbial networks, act as key regulators of community structure (e.g., microbial co-occurrence networks) and ecosystem functions (e.g., organic matter decomposition, nutrient cycling, and ecosystem stability) (Banerjee et al., 2018; Banerjee et al., 2019; Wang et al., 2023). A study on the assembly process of microbial communities on the Loess Plateau showed that keystone taxa played an important role in the assembly process of different vegetation restoration processes on the Loess Plateau (Shi et al., 2023); In addition, our previous study on an uninhabited mountain ultra-high altitude area in the southeast of the Qinghai Tibet Plateau also showed that certain microbial genera, even with low abundance, also largely drive the deterministic process of microbial assembly (Liu et al., 2025). These taxa also mediate interspecies interactions and enhance network resilience, but human disturbances can reduce their abundance, simplifying networks and amplifying dispersal limitations (Banerjee et al., 2019). For example, agricultural intensification diminishes root-associated keystone taxa, impairing ecosystem functions (Banerjee et al., 2018). Although extensive research links keystone taxa to ecosystem functioning, their role as mediators between human activities and microbial dynamics in sensitive ultra-high altitude regions, such as the Tibetan Plateau, remains unclear.

To address these gaps, this study selects 7 villages and 4 lands (4 areas with an area equal to the village), which across a 200 km transect in southeastern Tibet (Gongbo’gyamda County - Bayi District) for soil microbial community research, aiming to explore the effect of human activity on the assembly processes of soil microbial communities and the distribution of keystone taxa. Key research questions include: (1) How does increasing human activity intensity alter the balance between deterministic and stochastic processes in microbial community assembly? (2) To what extent are microbial dispersal processes restricted in regions with higher human disturbances? (3) How does human activity influence the distribution and abundance of keystone taxa? The research is essential for addressing global challenges such as biodiversity loss and soil degradation caused by human activities and will aid in the formulation of strategies for environmental protection and habitat restoration in the plateau region.

## 2 Materials and methods

### 2.1 Study area and soil sampling

**Figure 1:**
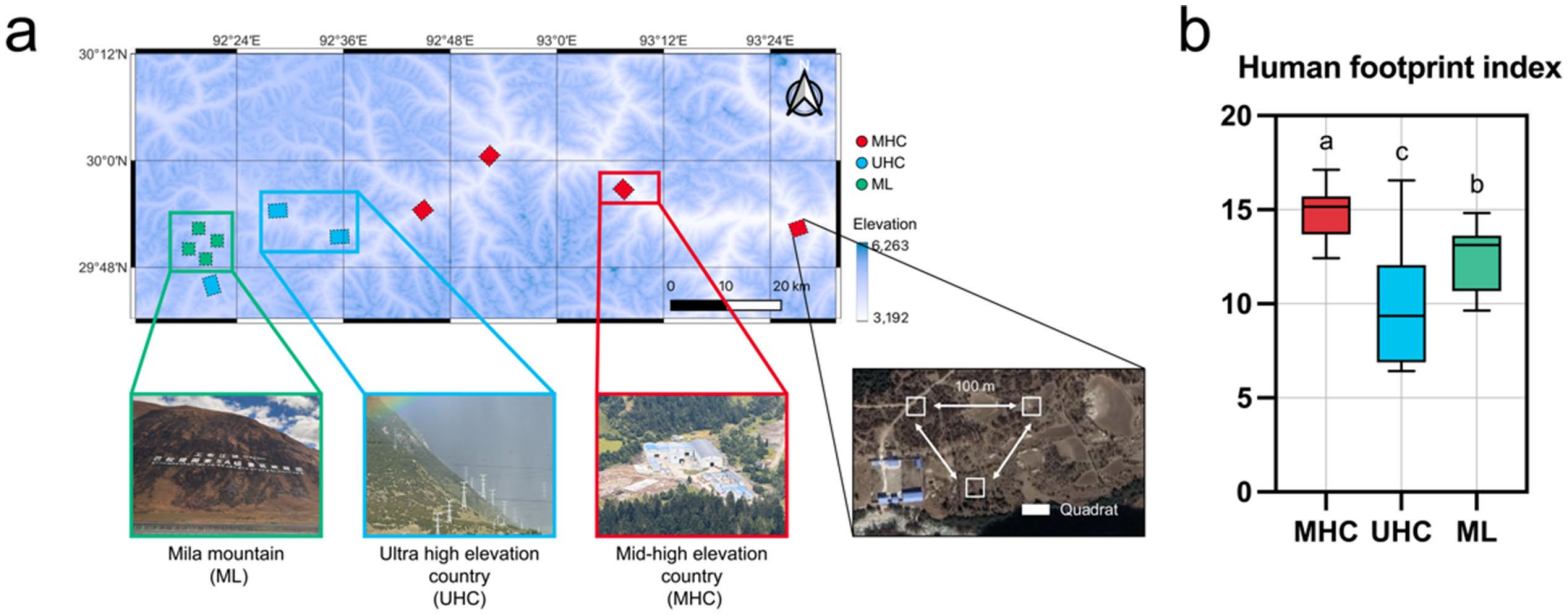
**(a)** Geographical locations of the sampling points with a landscape diagram of the three regions, and method of quadrats setting; **(b)** Differences in the Human Footprint Index (HFI) across the three regions, with significance comparisons conducted using the Kruskal-Wallis test (Bonferroni correction). Different letters represent significant differences (*p* < 0.05).

In October 3th, 2024, a field sampling was conducted across a 200 km transect from Gongbo’gyamda County to Bayi District in G318 highway. The G318 highway, one of China’s national road networks, passes through sparsely populated high-altitude villages, a national-level scenic area with high tourist and vehicle traffic (located in Gongbo’gyamda County’s Mila Mountain), and areas with higher human activity near urban zones in southeastern Tibet. A total of 11 sampling areas (1 km × 1 km, lands or villages) were categorized into three regions: Mila Mountain (ML), ultra-high elevation country (UHC), and mid-high elevation country (MHC). The average temperature of the sampling area was 5.005 °C, the average humidity was 82.40%, and the daily precipitation was 6.973 mm. All sampling areas had similar soil temperatures (average 7.576 °C) and humidity (average 39.53%), and were all conducted in primary grassland with vegetation coverage less than 50%. Each area contains three 10 m × 10 m quadrats, and quadrats were spaced more than 100 m apart from each other, which has a similar aspect and slope (Figure 1a). The details about sampling areas (names, geographical coordinates, elevations etc.) are summarized in Supplementary Table S1. Soil samples were collected from the surface layer (top soil, 0–20 cm) using a five-point sampling method in each quadrat. The five subsamples from each quadrat were combined into a single composite sample to minimize the heterogeneity of quadrats. All samples were transported to the laboratory with an ice pack immediately. Each mixed sample was then divided into two portions: one for soil physicochemical analysis and the other stored at −80 °C for subsequent DNA extraction.

Human footprint is the pressure exerted on the ecological environment by changing the ecological process and natural landscape, which has aroused the world’s attention to biodiversity and ecological protection. This study uses the Human Footprint Index (HFI) data published by Mu et al. (2022). In brief, Mu et al. use eight indicators (Built environment, Population density, Night-time lights, Croplands, Pasture, Poads, Railways, Navigable waterways) to reflect eight variables of different aspects of human stress. The results obtained from this data are in good agreement with previously developed datasets (Kennedy et al., 2019; Venter et al., 2016; Williams et al., 2020) of different years, so the data is credible. In this study, HFI data were extracted for each sampling plot using QGIS (V3.38, Free Software Foundation, Inc., Boston, MA), covering five time points: 2002, 2007, 2012, 2017, and 2022. The values were summarized according to the regional classifications (Figure 1b).

### 2.2 Soil chemical analysis and microbial sequencing

Soil chemical properties were analyzed to assess their influence on microbial community composition. The measured parameters included soil pH, total potassium (TK), total phosphorus (TP), soil organic carbon (SOC), total nitrogen (TN), available phosphorus (AP), available nitrogen (AN), and available potassium (AK), following the protocols described by Hou et al. (2024).

DNA extraction was performed using the FastDNA Spin Kit for Soil. Bacterial 16S rRNA genes were amplified using the primers 338F (5′-ATCCCTACGGGGGGGGGAGGCAG-3′) and 806R (5′-GGATTACHVGGGTWTCTAAT-3′), while fungal ITS regions were amplified with ITS1F (5′-CTGGTCATTTAGGGAAGTAA-3′) and ITS2R (5′-GTGCGTTCTTCATCGATGC-3′). Sequencing was conducted on the Illumina MiSeq PE300/NovaSeq PE250 platform by Majorbio Bio-Pharm Technology Co., Ltd., Shanghai, China. The raw sequences were processed using QIIME2, with denoising and amplicon sequence variant (ASV) clustering performed via the DADA2 pipeline. Taxonomic assignment was carried out using the Silva database (16S/silva_de_uncultured_All/v132) for bacterial sequences and the UNITE database (ITS/unite_v8.0_de_unidentified_All/v8.0) for fungal sequences. The raw sequencing data have been deposited in the National Center for Biotechnology Information (NCBI) under BioProject accession number PRJNA1228301.

### 2.3 Microbial community assembly processes

A maximum-likelihood phylogenetic tree was constructed for bacterial 16S rRNA sequences using FastTree (Price et al., 2010). Due to the challenges associated with building robust phylogenetic trees for highly variable marker genes like ITS (Ning et al., 2020), fungal phylogenies were inferred using the taxonomy_to_tree.pl. script in Perl (V5.40.0.1) (Tedersoo et al., 2018).

Microbial community assembly processes were analyzed using the R package “iCAMP” (V1.5.12), which applies a null model framework with 1,000 times randomizations. This randomization procedure repeatedly shuffles the phylogenetic relationships and species occurrence data across phylogenetic bins under a specific null hypothesis, generating a distribution of expected ecological patterns in the absence of a given assembly process (Ning et al., 2020). iCAMP categorizes microbial community assembly into five ecological processes: deterministic [heterogeneous selection (HeS), homogeneous selection (HoS)] and stochastic [dispersal limitation (DL), homogeneous dispersal (HD), and drift (DR)] (Ning et al., 2020). Finally, a total of 35,596 bacterial ASVs and 8,307 fungal ASVs were binned into 644 and 196 phylogenetic bins, respectively. Phylogenetic trees were constructed using a modified taxonomy_to_tree.pl. script, which remapped biological classifications, making the binning results reliable (Ning et al., 2020).

### 2.4 Statistical analysis

Microbial alpha diversity, principal coordinates analysis (PCoA) based on Bray-Curtis distance and variance decomposition analysis (VPA) were calculated using the “vegan” package (V2.6-8) (Oksanen et al., 2024). The “linkET” package (V0.0.7.4) (Huang, 2021) was used to conduct a Mantel-test for microbial diversity and soil essential chemical factors. Microbial co-occurrence network analysis was conducted using the “igraph” (V2.1.2) and “Hmisc” (V5.2-1) packages (Harrell, 2024), including taxa with relative abundances > 0.01%. The “randomForest” package (4.7-1.2) was used for microbial classification prediction (Liaw and Wiener, 2002), and the “mixOmics” package (6.28.0) was used to compute variable importance in projection (VIP) values (Rohart et al., 2017). Keystone taxa were identified based on the following criteria: high degree centrality in the co-occurrence network, VIP > 1, top 1% mean decrease accuracy (MDA) values, and > 60% prevalence. Spearman correlation analyses were performed between keystone taxa, soil physicochemical properties, HFI, and microbial community assembly processes using the “pheatmap” package (V1.0.12) (Kolde, 2019). A partial least squares path model (PLS-PM) was constructed using the “plspm” package (V0.5.1) (Sanchez et al., 2024). Data of DR were conducted “× −1” conversion to ensure model correctness. All data incorporated into the path model underwent SAR residualization and Moran’s I test using the R package “spdep.” All packages were run in R (V4.4.1, R Core Team, 2024). All statistical significance tests were conducted in SPSS (V26.0, IBM, Armonk, NY, United States). The visualization of the co-occurrence network was generated using Gephi (V0.10.1), and the visualization of the remaining data was done by Graphpad Prism (V10.3.0, GraphPad Software, San Diego, CA, United States) and the “ggplot2” package (Wickham, 2016).

## 3 Results

### 3.1 Human activity intensity and changes in soil chemical properties

The MHC exhibited the highest human activity intensity (HFI = 14.77), while the UHC had the lowest (HFI = 9.88). The human activity intensity in the ML (HFI = 12.55) was between the two previously mentioned regions (Figure 1b). ML had the lowest SOC (38.10 g/kg), while UHC had the lowest AN (281.16 mg/kg), but UHC had the highest AK (308.98 mg/kg) and AP (476.87 mg/kg). The soil pH of the three regions was similar, all of them were acidic soil (MHC: 6.52; UHC: 6.57; ML: 6.17) (Supplementary Figure S1). In addition, human activities increased soil AN (*r* = 0.50), but decreased the AP (*r* = −0.46) directly (*p* < 0.05), While elevation decreased AK (*r* = −0.70) (Supplementary Figure S2).

### 3.2 Basic information of microorganisms

**Figure 2:**
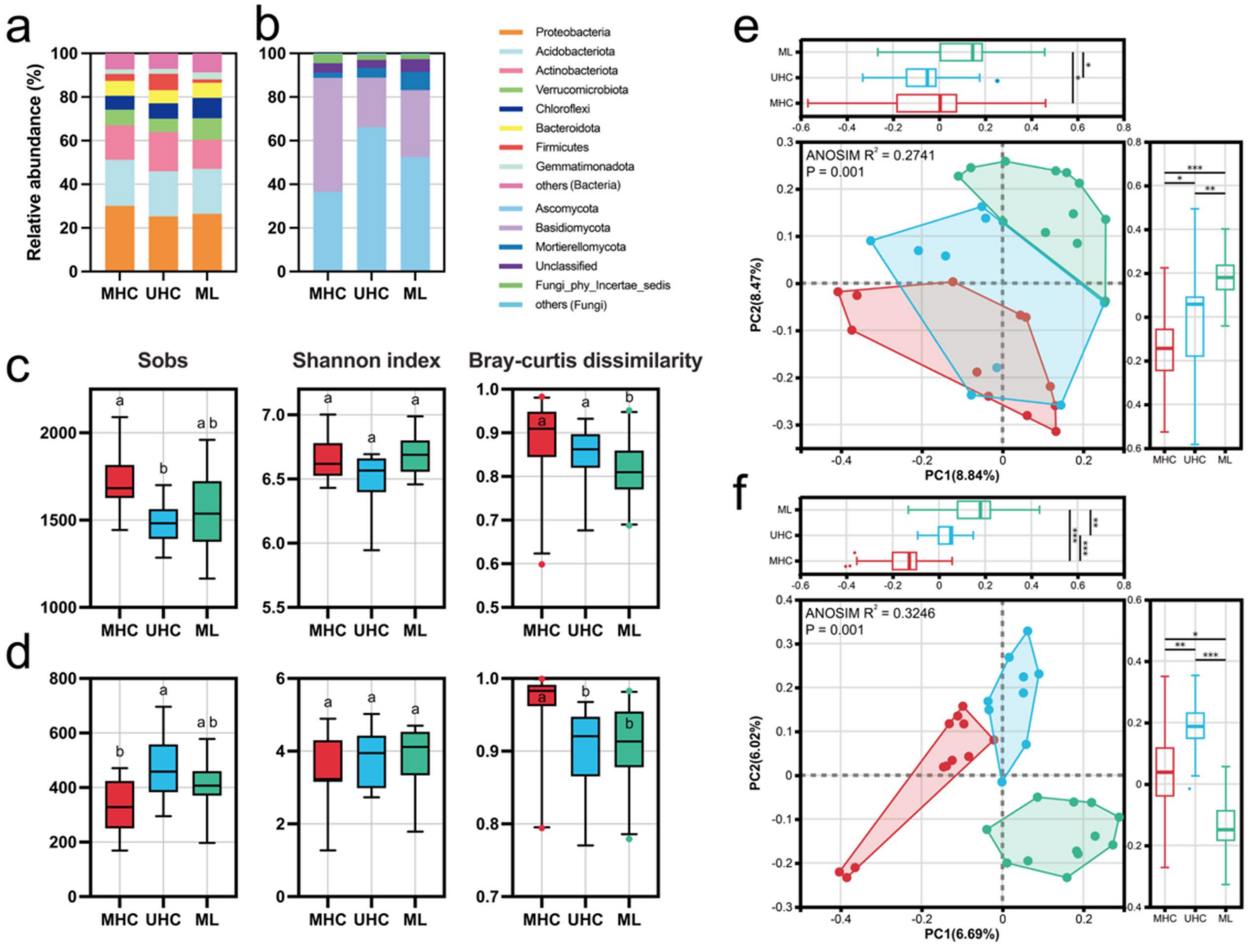
Microbial basic information, including: Relative abundance of **(a)** bacteria and **(b)** fungi in different regions; Observed ASV numbers (Sobs), Shannon index, and Bray–Curtis dissimilarity of **(c)** bacteria and **(d)** fungi in different regions [Significance comparisons were conducted using the Kruskal-Wallis test (Bonferroni correction)]; Principal coordinate analysis (PCoA) results for **(e)** bacteria and **(f)** fungi in different regions. Different letters representing significant differences (*p* < 0.05), and asterisks indicating significant differences (*p < 0.05; **p < 0.01; ***p < 0.001). MHC, mid-high elevation country; UHC, ultra-high elevation country; ML, Mila mountain.

At the phylum level, the relative abundance of bacterial phyla showed little difference across the three regions, with Proteobacteria, Acidobacteria, Actinobacteria, Verrucomicrobiota, and Chloroflexi dominated the bacterial communities (MHC: 80.55%; UHC: 77.17%; ML: 79.59%) in each region (Figure 2a). Ascomycota and Basidiomycota dominated the fungal communities (MHC: 88.78%; UHC: 88.92%; ML: 83.14%). In MHC, Basidiomycota had a higher relative abundance than Ascomycota, while in UHC and ML, Ascomycota had a higher relative abundance (Figure 2b).

Bacteria showed higher richness and diversity indices overall compared to fungi. The richness of both bacteria and fungi differed significantly between regions, with opposite trends observed for bacteria and fungi across the regions (*p* < 0.05) (Figures 2c,d). Using Bray-Curtis distance as an indicator of microbial community structure, it was found that in MHC, bacterial and fungal community structures usually showed higher differences (Figures 2c,d). However, some previous studies showed that the increase of geographical distance will increase beta diversity (i.e., distance-decay relationship, Martiny et al., 2011; Wang et al., 2017). Therefore, we selected two villages with the highest HFI and the closest distance in the MHC (Langsha and Binge) for re-statistics and found no statistically different from the previous results, indicating that distance may not be the main factor dominating the beta diversity variation in our study. Correlation analysis indicates that the alpha diversity of bacteria is more influenced by nitrogen (for Sobs, *r* = 0.37) and phosphorus (for Sobs, *r* = −0.41, for Shannon index, *r* = −0.37) than that of fungi. Human activites increased bacterial alpha diversity but decrease fungal, although they were not significant (Supplementary Figure S2). Generally, human activities could change soil physical and chemical properties, then influence the richness (Sobs) and diversity (Shannon index) of bacteria and fungi. Moreover, although there is no consistent linear pattern between microbial composition and diversity and altitude (Supplementary Figure S3), previous studies have shown that the composition of soil microbes in alpine regions is more driven by soil properties than just altitude (Chen et al., 2025; Fu B. et al., 2023; Fu F. et al., 2023).

Principal coordinates analysis (PCoA) of beta diversity further revealed differences between regions. ANOSIM R (bacteria: 0.2741; fungi: 0.3246) and *p*-values (*p* < 0.001) indicated that the microbial community structure of the three regions was significantly different (Figures 2e,f).

### 3.3 Community assembly processes analysis

**Figure 3:**
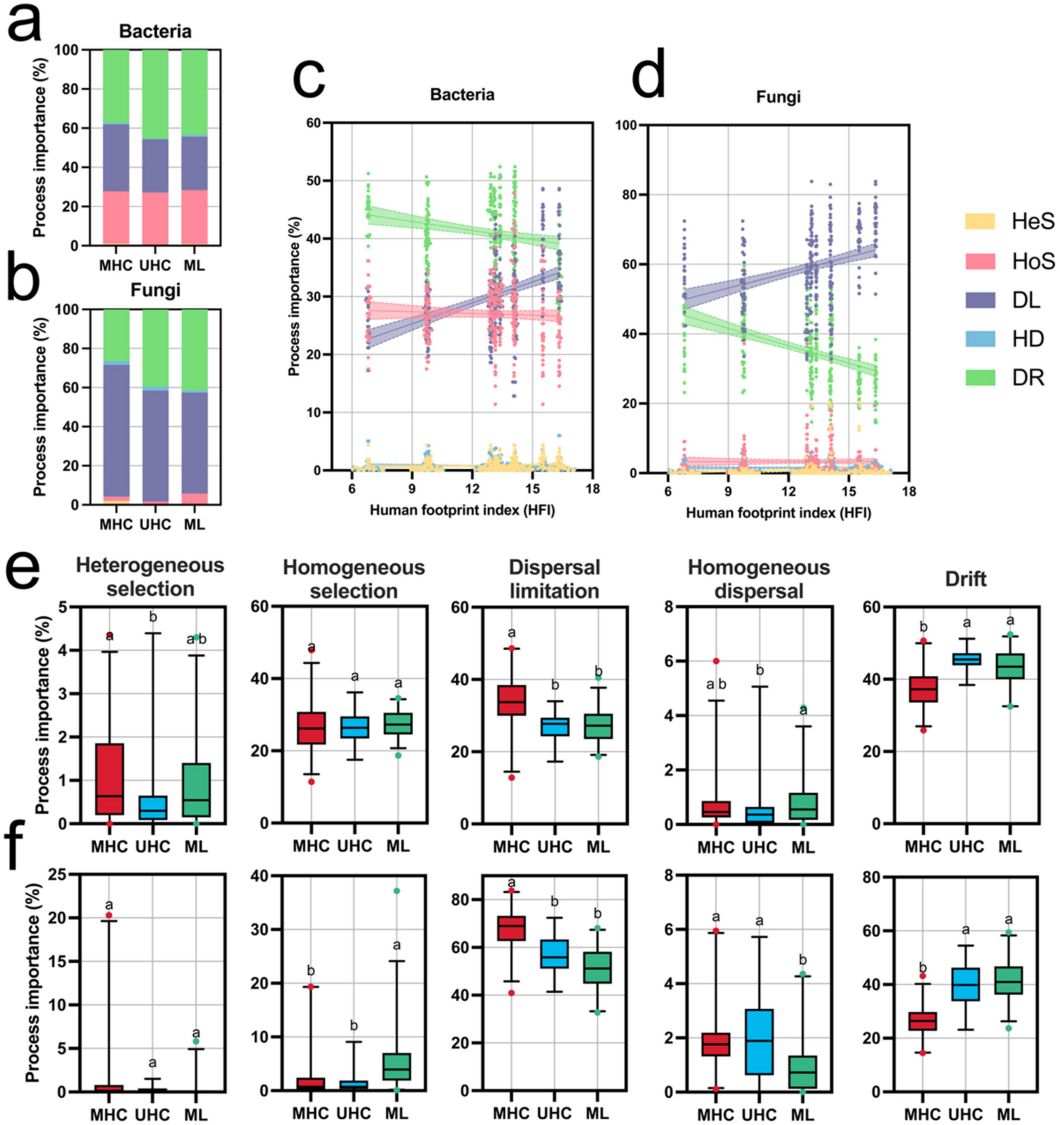
Results of microbial community assembly process analysis using the iCAMP package, including: Proportions of the five ecological processes for **(a)** bacteria and **(b)** fungi in different regions; Changes in the relative importance of the five ecological processes for **(c)** bacteria and **(d)** fungi as the Human Footprint Index (HFI) changes (linear regression results are shown in Supplementary Table S2); Differences in the relative importance of the five ecological processes for **(e)** bacteria and **(f)** fungi in different regions. Significance comparisons were conducted using the Kruskal-Wallis test (Bonferroni correction), with different letters representing significant differences (*p* < 0.05). MHC, mid-high elevation country; UHC, ultra-high elevation country; ML, Mila mountain; HeS, heterogeneous selection; HoS, homogeneous selection; DL, dispersal limitation; HD, homogeneous dispersal; DR, drift. Deterministic processes including HeS, HoS, while stochastic processes including DL, HD, and DR.

iCAMP results showed that the assembly process of both bacteria and fungi was primarily driven by stochastic processes (bacteria: MHC: 72.27%; UHC: 72.82%; ML: 71.59%. fungi: MHC: 95.78%; UHC: 98.28%; ML: 94.22%). In bacteria, the relative importance of DR (MHC: 37.27%; UHC: 45.22%; ML: 43.44%) was higher than that of DL (MHC: 34.24%; UHC: 27.09%; ML: 27.20%), while in fungi, DL (MHC: 67.37%; UHC: 56.73%: ML: 51.64%) was more important than DR (MHC: 26.50%; UHC: 39.60%; ML: 41.51%, indicating that bacteria were influenced by more unobservable processes). Additionally, HoS (MHC: 26.69%; UHC: 26.62%; ML: 27.54%) played a more significant role in bacterial community assembly (Figures 3a,b).

Human activites influenced assembly processes significantly. High levels of human activities limit the dispersal of communities, as well as reducing the process importance of drift. In both bacteria and fungi, DL was positively correlated with HFI (*p* < 0.0001), showing a strong positive association, while DR showed the opposite pattern (*p* < 0.0001) (Figures 3e,f; Supplementary Table S2). Significant differences in community assembly processes were observed among three regions: MHC had a significantly higher HeS for bacteria compared to UHC, while HoS for fungi was significantly higher in ML than in MHC and UHC. DL for both bacteria and fungi in MHC was significantly higher than in UHC and ML, while DR exhibited the opposite trend (*p* < 0.05). However, the importance of HoS in bacterial community assembly did not show significant differences (Figures 3c,d). However, assembly processes shows nonlinear changes with altitude, which is particularly obvious in bacteria, presenting a horseshoe-shaped pattern (DL and DR) (Supplementary Figure S4).

### 3.4 Co-occurrence network analysis and keystone taxa screening

**Figure 4:**
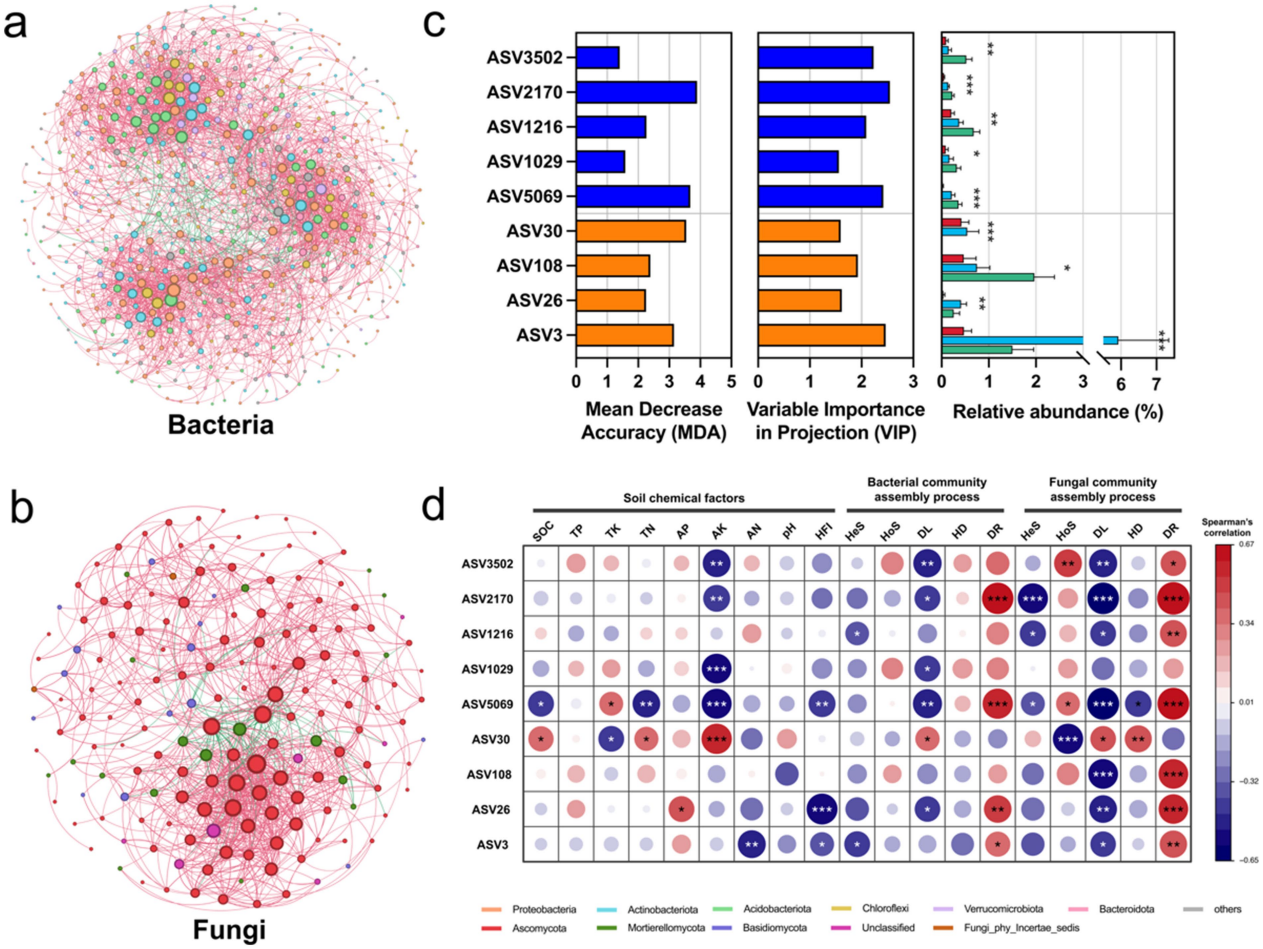
**(a)** Bacterial and **(b)** fungal co-occurrence network analysis results (detailed network indicators are shown in Supplementary Table S3); **(c)** Keystone taxa selected using random forest and PLS-DA, with blue representing bacteria and orange representing fungi. The bar chart on the right shows the relative abundance differences of keystone taxa in different regions (Red: MHC; Light blue: UHC; Green: ML). Significance comparisons were conducted using the Kruskal-Wallis test (Bonferroni correction), with asterisks indicating significant differences (*p < 0.05; **p < 0.01; ***p < 0.001); **(d)** Spearman correlation analysis between keystone taxa and soil chemical factors, bacterial and fungal community assembly processes, with asterisks indicating significant correlations (*p < 0.05; **p < 0.01; ***p < 0.001).

In the bacterial network, MHC exhibited the highest number of nodes (760) and edges (16934), with the lowest modularity (0.5530) among three regions. UHC, although having the highest number of nodes (1026), had significantly lower edges (9665) than MHC. ML had the lowest number of nodes (588) and edges (1043), but had the higest modularity (0.9277). In the fungal network, UHC showed the highest nodes (328) and edges (1196), while MHC exhibited the lowest modularity (0.6797) (Supplementary Table S3). In addition, compared with the ALL group (integrating all groups), the bacterial ALL group had significantly more nodes (585) and edges (3013) than the fungal network (nodes: 171; edges: 921), but the fungal ALL group exhibited higher network density (0.0634) and clustering coefficient (0.4859) than the bacterial network (density: 0.0176; clustering coefficient: 0.3330), suggesting that fungi might form tighter and more locally clustered interaction patterns in the overall network (Supplementary Table S3; Figures 4a,b). In general, human activities, which generated interference, increased the complexity of the network but decreased its stability.

Subsequently, keystone taxa were selected based on node degree in the ALL group network, combined with MDA and VIP value (Supplementary Table S4). It was found that the relative abundance of keystone taxa varied significantly across regions (*p* < 0.05), with keystone bacteria generally showing an increasing trend in abundance from MHC to UHC to ML (Figure 4c). It suggests that high intensity human activities (in MHC) led to the loss of keystone taxa.

Spearman correlation analysis of keystone taxa with soil chemical properties, HFI, and community assembly processes revealed that keystone taxa were correlated with all factors except TP, pH, and bacterial HoS and HD. Particularly strong correlations were found between keystone taxa and AK, DL, DR, fungal HoS (*p* < 0.05). Most of keystone taxa were negatively correlated with HFI (Figure 4d).

### 3.5 Factors influencing community assembly processes

**Figure 5:**
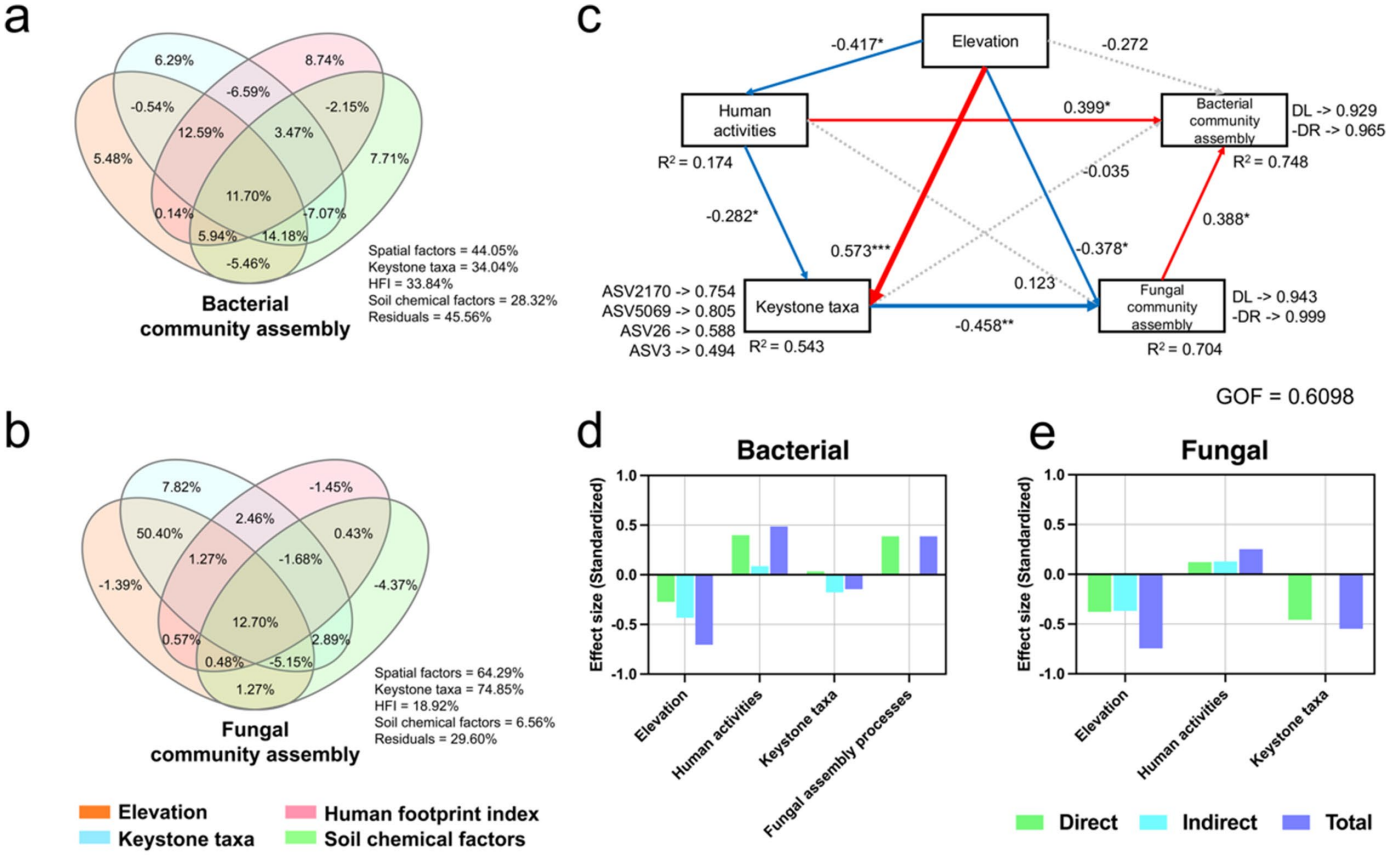
Variance partitioning analysis (VPA) was used to show the factors affecting the assembly process of bacterial **(a)** and fungal **(b)** communities. **(c)** The associations between elevation, human activities, keystone taxa, and community assembly processes based on the Partial Least Squares Path Model (PLS-PM); The effect size of elevation, human activities, and keystone taxa on bacterial **(d)** and fungal **(e)** community assembly processes. All data included in PLS-PM passed Moran’s I test (*p* > 0.05) to verify whether there were spatial differences.

Although Supplementary Figure S3 has indicated a weak correlation between basic information of microbial communities and altitude, the assembly process presented in Supplementary Figure S4 still follows a horseshoe-shaped pattern with altitude. Therefore, we used variance partitioning analysis to explore the effects of elevation, HFI, keystone taxa, and soil physicochemical properties on assembly process (Figures 5a,b). The results showed that both HFI (33.84%) and keystone taxa (34.04%) had a high explained variation for the bacterial assembly process, with keystone taxa being more important for the fungal assembly process (74.85%) and HFI having a relatively lower explained variation for fungi (18.92%). The influence of physicochemical properties on the assembly process was generally minor (bacteria: 28.32%; fungi: 6.56%). Furthermore, elevation still had an explained variation for the assembly process that cannot be underestimated (bacteria: 44.05%; fungi: 64.29%).

We further using Partial Least Squares (PLS) to construct a path model (Figure 5c–e, Moran’s I test results in Supplementary Table S5), the results indicated that human activities contributed to the loss of keystone taxa (*r* = −0.282, *p* < 0.05) and exacerbated dispersal limitation of microbial communities (bacteria: 0.488; fungi: 0.252), with a direct impact on bacteria (*r* = 0.399, *p* < 0.05). However, the direct impact of human activities on the fungal assembly process was not significant, but it may hinder fungal dispersal by reducing keystone taxa (*r* = −0.458, *p* < 0.01). Overall, as elevation increases, the dispersal of microbial communities becomes prevalent. However, human activities, either directly or indirectly (e.g., by reducing keystone taxa), affect this process, exacerbating the limitations on dispersal.

## 4 Discussion

### 4.1 The impact of human activities on microbial community structure in ultra-high altitude regions

The relationship between human activities and soil microbial ecology has been a key topic in environmental ecology. Soil microbial community structures and assembly processes reflect environmental changes. Extensive human activities, such as grazing and tilling, alter natural environments by causing soil compaction and habitat fragmentation, significantly impacting microbial communities (Niu et al., 2024; Zhang et al., 2025). Most previous research has concentrated on urban regions, but the once sparsely populated Tibetan Plateau, particularly ultra-high-altitude areas, now faces increasing pressures due to China’s rapid development. This study reveals strong direct and indirect impacts of human activities on soil microbial communities in ultra-high-altitude regions of the Tibetan Plateau, providing a basis to further investigate microbial responses to human disturbances in ecologically sensitive regions.

Human activities significantly affected the nitrogen and phosphorus content in the three regions, which in turn affected the richness and diversity of microbial communities. (Figures 2c,d; Supplementary Figure S2). Microbes are more dependent on soluble nitrogen sources (such as ammonium and nitrate) (Farrell et al., 2014). In regions with high HFI, human activities (e.g., grazing, tilling, fertilizing) can lead to nitrogen enrichment, but decrease phosphorus due to overfarming (Supplementary Figures S1, S2), which promotes microbial growth under conditions of sufficient nitrogen. However, human activities (e.g., plowing) cause soil disturbance, which disrupts fungal mycelial networks (Anderson et al., 2017; Emilia Hannula and Morriën, 2022), leading to a reduction in fungal abundance. In contrast, bacteria, with their smaller size and more diverse forms of presence in the soil, are better able to survive and reproduce under a variety of soil conditions, including those affected by human disturbance. Similarly, human activities increase environmental heterogeneity at different locations, thus promoting the heterogeneity of microbial communities (Li et al., 2021). Our results also reflect this (Figures 2c,d). In addition, human activities also influenced microbial networks, which increased the complexity of microbial networks, but decreased the stability of the system (Supplementary Table S3). There is no simple one-to-one correspondence between network complexity (such as the number of nodes, edges, and average degree) and stability; stability depends on the type of interaction (competition, predation, mutualism), the distribution of interaction strength, modularity, and the positive/negative ratio (May, 1972). Tight coupling (high connectivity) may lead to network fragility or collapse, especially when many positive relationships exist; conversely, high modularity or more negative relationships can improve the system’s fault tolerance and persistence to local perturbations (Coyte et al., 2015; Faust and Raes, 2012) The higher modularity indicates a stable network by limiting the loss of taxonomic units’ impact on the community (Shi et al., 2023). MHC has the highest number of network nodes and edges, yet it has the lowest modularity.

Although our results highlight the impact of human activities on the composition and diversity of microbial communities, our 11 sampling areas do indeed have an altitude gradient. However, we unexpectedly found that the composition and diversity of microbial communities did not show regular changes with altitude (Supplementary Figures S3, S4). Previous studies in the Qinghai-Tibet Plateau or other mountainous regions (e.g., Qilian Mountains) have also shown that the composition and diversity of microorganisms are more determined by soil physical and chemical properties and nutrient supply rather than altitude itself (Fu B. et al., 2023; Wang et al., 2020; Yang et al., 2014). The latest study on the vertical distribution pattern of soil microorganisms in the alpine meadow region of the Qinghai-Tibet Plateau also indicates that the top soil does not show significant a monotonous or linear tendency to change with altitude changing (Chen et al., 2025).

### 4.2 Human activities affect community assembly processes and keystone taxa distribution in ultra-high altitudes

Elucidating microbial assembly processes is crucial for understanding ecosystem diversity and function (Zhou and Ning, 2017). Our study shows that stochastic processes dominate the community assembly process across different regions (Figures 3a,b). This result contrasts sharply with findings from urban ecosystems in more developed provinces such as Guangdong (Fu F. et al., 2023). In urban areas experiencing intense urbanization and industrialization, deterministic processes typically dominate due to severe environmental pollution (Fu F. et al., 2023). Zhang et al. (2022) indicated that high pollutant concentrations enhance deterministic assembly processes. However, human activities in ultra-high-altitude regions mainly involve agricultural practices such as grazing rather than urban expansion, resulting in relatively lower pollution levels and consequently less pronounced deterministic effects. Additionally, microorganisms in areas with human activities might have adapted to these dynamic environments, reducing their sensitivity to environmental variations and resulting in very low heterogeneous selection across all regions (Liu et al., 2020; Chen et al., 2019).

We also found that the higher the HFI, the more significant the role of dispersal limitation in the community assembly process for both bacteria and fungi (Figures 3c,d; Supplementary Table S2). Fungi experienced stronger dispersal limitations than bacteria, likely due to their generally larger body sizes (Farjalla et al., 2012). Human activities likely enhance spatial isolation of microorganisms primarily by disrupting microbial dispersal routes—such as fungal mycelial networks or bacterial biofilms—or by altering soil physicochemical properties, thereby influencing dispersal efficiency (Emilia Hannula and Morriën, 2022; Anderson et al., 2017; Tripathi et al., 2018). Human activities in ultra-high-altitude areas typically increase habitat heterogeneity (e.g., trampled vs. non-trampled areas, grazed vs. non-grazed areas), intensifying spatial isolation and further strengthening dispersal limitation processes (Hanson et al., 2012; Banerjee et al., 2019). In addition, variance partitioning analysis revealed that elevation contributed substantially to assembly processes (Figures 5a,b), often promoting dispersal as altitude increases, due to greater environmental homogeneity or reduced filtering (Yang et al., 2014; Chen et al., 2025). However, HFI and keystone taxa also explained high variation, indicating human disturbances override altitudinal effects.

Keystone taxa are critical for understanding the stability of complex microbial assemblages. They provide essential driving forces in shaping the structure and functioning of microbial communities (Banerjee et al., 2018). Our study highlights keystone taxa as pivotal links between human activities and microbial community dynamics (Figures 5a,b). The relative abundances of most keystone taxa were significantly suppressed by human activities (Figure 4d), and these taxa exhibited significant negative correlations with dispersal limitation (Figures 5c–e), likely serving as bridges that facilitate microbial interactions and dispersal. However, these key microorganisms were notably reduced in abundance within MHC (Figure 4c), exacerbating dispersal limitations and potentially increasing microbial network instability. *Sphingomonas* (ASV5069), known for its capacity to degrade organic pollutants such as polycyclic aromatic hydrocarbons (Song et al., 2022; Sharma et al., 2021), was expected to exhibit higher abundance in regions of intensified human activity, yet our results indicated the opposite (Figure 4c), with the lowest abundance observed in MHC. Similarly, *Gaiella* (ASV2170), involved in nitrogen cycling and redox processes (Zhou et al., 2017), was least abundant in MHC (Figure 4c), despite higher AN levels there. Although keystone taxa abundances declined in MHC, microbial communities could employ functional redundancy to maintain overall microbial functions, partially preserving community stability (Peng et al., 2024). Nevertheless, such redundancy might reduce microbial resilience, and excessive redundancy could ultimately destabilize the community (Fan et al., 2025). Overall, the loss of these taxa might weaken soil functions, diminish environmental disturbance response capabilities, and further reduce microbial community resilience in MHC.

Moreover, it is worth emphasizing that ultra-high altitude ecosystems are particularly sensitive to human activities compared to lower altitudes (Mao et al., 2023). The short growing season and slow physiological and decomposition processes limit natural recovery capacity (Tian et al., 2023). Furthermore, these ecosystems exhibit low functional redundancy, relying on few key functional groups for carbon and nitrogen cycles, making keystone taxa reductions cause more significant functional losses. The superimposed effects of localized pressures (e.g., roads, tourism, grazing) with altitude and climate change amplify ecological consequences, necessitating more cautious and targeted management than in low-altitude areas (Che et al., 2025).

In summary, in the ultra-high altitude regions of the Qinghai-Tibet Plateau, human activities have significantly altered the nitrogen and phosphorus contents of the soil, thus influencing microbial community richness and diversity as well as the assembly process mediated by keystone taxa. Elevation promotes microbial dispersal, but human activities directly or indirectly suppress this effect, as evidenced by PLS-PM and VPA results. In addition, human activities reduced keystone taxa and destroyed habitats to inhibit the dispersal of communities directly. The observed loss of keystone taxa provides important indicators of ecosystem instability, underscoring their value in ecological assessment and conservation.

## 5 Conclusion

This study demonstrates that human activities significantly alter soil nutrient dynamics and influence microbial diversity and community assembly processes in ultra-high altitude regions of the southeastern Tibetan Plateau. Our results indicate that heightened human disturbance exacerbates dispersal limitation for both bacterial and fungal communities, while simultaneously reducing the abundance of keystone taxa that are vital for microbial network stability. Notably, keystone taxa such as *Sphingomonas* and *Gaiella* were negatively correlated with human activity intensity, suggesting their sensitivity to anthropogenic pressure. Although elevation generally promotes microbial dispersal, human activities directly and indirectly counteract this effect by inducing habitat fragmentation and reducing keystone taxa prevalence. These findings underscore that human activities not only modify soil properties and microbial diversity but also restructure assembly processes and interaction networks, ultimately increasing ecological vulnerability. Therefore, effective conservation measures should focus on minimizing anthropogenic nutrient inputs and preserving microbial keystone taxa to enhance ecosystem resilience in these fragile high-altitude regions.

## Data Availability

The datasets presented in this study can be found in online repositories. The names of the repository/repositories and accession number(s) can be found in the article/Supplementary material.
