# Supplementary material for: High intensity human activity limits ultra-high altitude soil microbial community dispersal and keystone taxa distribution in southeastern Tibetan Plateau
Source: Front Microbiol. 2025 Oct 3;16:1666493. doi: 10.3389/fmicb.2025.1666493 (PMC12531163; doi:10.3389/fmicb.2025.1666493)
Supplement: Supplementary file 2 [file Data_Sheet_1.docx]

**High intensity human activity limits ultra-high altitude soil microbial community dispersal and keystone taxa distribution in southeastern Tibetan Plateau**

Wenzu Liu^1,2,#^, Hongfang Ma^2,#^, Mengyao Dong^1,2^, Yaning Song^3^, Ruihong Wang^1^, Zhuonan Hou^4,5^, Daqing Luo^1^, Heping Ma^1,*^, Yuquan Wei^1,2,*^

*^1^ Institute of Tibet Plateau Ecology, Xizang Agricultural and Animal Husbandry University, Nyingchi, China*

*^2^ College of Resources and Environmental Science, Beijing Key Laboratory of Biodiversity and Organic Farming, China Agricultural University, Beijing, China*

*^3^ College of grassland science and technology, China Agricultural University, Beijing, China*

*^4^ State Key Laboratory for Soil Erosion and Dryland Farming on the Loes Plateau, Institute of Soil and Water Conservation, Chinese Academy of Science and Ministry of Water Resources, Yangling, Shaanxi, China*

*^5^ University of Chinese Academy of Sciences, Beijing, China*

* Corresponding author

E-mail addresses: weiyq2019@cau.edu.cn (Y. Wei); maheping@xza.edu.cn (H. Ma)

# Authors contributed equally to this work

Supporting Information Includes:

- 5 figures

- 5 tables

Figure S1. Soil basic physical and chemical factors in each region, with significance comparisons conducted using the Kruskal-Wallis test (Bonferroni correction). Different letters represent significant differences (*p* < 0.05). MHC: mid-high elevation country; UHC: ultra-high elevation country; ML: Mila Mountain.

Figure S2. (a) Bray-Curtis dissimilarity of bacteria and fungi in different regions. (b) Spearman correlation analysis of soil basic physicochemical factors, HFI & elevation and microbial diversity.

Figure S3. Composition, abundance, and diversity of bacteria and fungi at different altitudes.

Figure S4. Nonlinear fitting result of microbial community assembly processes with altitudes.

Figure S5. Prediction accuracy of (a) bacteria and (b) fungi using random forest algorithm. MHC: mid-high elevation country; UHC: ultra-high elevation country; ML: Mila Mountain.

Supplementary tables were detailed in the Excel documentation.

Table S1. Sampling area details of three regions.

Table S2. Linear regression results of different ecological processes of community assembly with Human Footprint Index (HFI).

Table S3. Detailed results of microbial co-occurrence network analysis.

Table S4. Taxonomy for keystone taxa.

Table S5. Moran’s I test result of data in PLS-PM.
